# Investigation of Sensitivities and Drift Effects of the Arrayed Flexible Chloride Sensor Based on RuO_2_/GO at Different Temperatures

**DOI:** 10.3390/s18020632

**Published:** 2018-02-20

**Authors:** Shi-Chang Tseng, Tong-Yu Wu, Jung-Chuan Chou, Yi-Hung Liao, Chih-Hsien Lai, Siao-Jie Yan, Ting-Wei Tseng

**Affiliations:** 1 Graduate School of Mechanical Engineering, National Yunlin University of Science and Technology, Douliu 64002, Taiwan; E-Mail: tsengsc@yuntech.edu.tw (S.-C.T.); d10011005@yuntech.edu.tw (T.-Y.W.); 2 Department of Electronic Engineering, National Yunlin University of Science and Technology, Douliu 64002, Taiwan; E-mail: chlai@yuntech.edu.tw (C.-H.L.); wolf84411@gmail.com (T.-W.T.); 3 Graduate School of Electronic Engineering, National Yunlin University of Science and Technology, Douliu 64002, Taiwan; E-mail: m10313322@yuntech.edu.tw; 4 Department of Information and Electronic Commerce Management, TransWorld University, Douliu 64002, Taiwan; E-mail: liaoih@mail.twu.edu.tw

**Keywords:** ruthenium dioxide, chloride ion sensor, graphene oxide, temperature effect, drift effect

## Abstract

We investigate the temperature effect on sensing characteristics and drift effect of an arrayed flexible ruthenium dioxide (RuO_2_)/graphene oxide (GO) chloride sensor at different solution temperatures between 10 °C and 50 °C. The average sensor sensitivities according to our experimental results were 28.2 ± 1.4 mV/pCl (10 °C), 42.5 ± 2.0 mV/pCl (20 °C), 47.1 ± 1.8 mV/pCl (30 °C), 54.1 ± 2.01 mV/pCl (40 °C) and 46.6 ± 2.1 mV/pCl (50 °C). We found the drift effects of an arrayed flexible RuO_2_/GO chloride sensor in a 1 M NaCl solution to be between 8.2 mV/h and 2.5 mV/h with solution temperatures from 10 °C to 50 °C.

## 1. Introduction

Many researchers have investigated the sensitivity, response time and drift rate of chloride ion sensing devices, but few researchers have studied the effect of temperature effect on their chloride ion sensing devices. However, the sensitivities and longer period detecting chloride ion concentrations of the chloride ion sensing devices are interesting subjects for study at different solution temperatures. Temperature affects the sensitivity of pH sensors, and many researchers have investigated sensitivity variation with pH solution temperatures from 25 °C to 65 °C [1,2,3,4]. They found that pH sensitivity increased as solution temperature increased. They calculated the temperature coefficient of sensitivity (TCS) for the pH sensors, and investigated the relationship between TCS and pH sensors. Many researchers used a radio frequency (RF) sputtering system [5,6,7] and screen printing technology [8] to fabricate the RuO_2_ sensing electrode. They have investigated and applied the physical characteristic of ruthenium.

**Figure 1:**
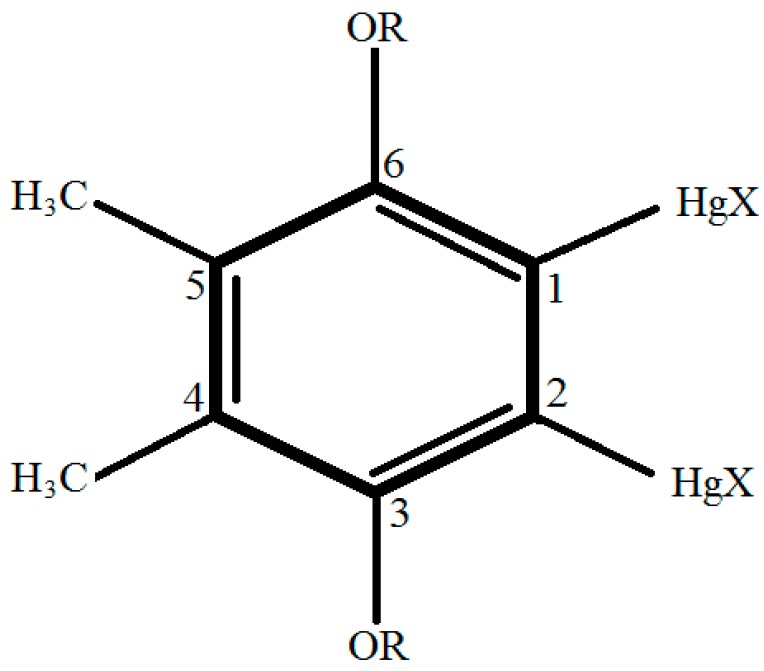
The mercury organic ionophore structure (ETH9033) [10].

Poly (vinyl chloride) (PVC), bis (2-ethylhexyl) sebacate (DOS), chloride ionophore III (ETH9033) and tridodecylmethy-lammonium chloride (TDDMACl) were used to fabricate the chloride sensing films for different chloride sensors [9,10,11,12,13,14]. The chemical reaction process when exposed to chloride ions, is as shown in (1) [10]. Figure 1, Ar–Hg–R depicts a mercury organic compound, and X depicts chloride ion.

Ar–Hg–R + X^−^ ↔ Ar–Hg(–R)−X^−^ ↔ Ar–Hg–X–R^−^ (1)

Our research group used the arrayed flexible RuO_2_ chloride sensor to investigate real applications for tap water and swimming pool water [14]. The response potentials of the arrayed flexible RuO_2_ chloride sensor were −245.711 ± 1.410 mV (0.248 mg/L chloride concentration) and −256.058 ± 2.097 mV (0.998 mg/L chloride concentration), respectively, for tap water and swimming pool water. Mahajan et al. [15] employed Cu(II) complexes to develop highly sensitive and selective chloride sensors, sensing across chloride concentrations ranging from 2.5×10^−5^ M to 1.0×10^−1^ M. Garrido et al. [16] used screen printing to fabricate the three electrodes of a wearable electrochemical sensor. The sensing detection limit was 2.0 × 10^−4^ M for chloride ions. Montemor et al. [17] fabricated a multi-probe chloride sensor and used it to measure response potentials of a mortar and concrete specimen. Trnkova et al. [18] prepared a carbon paste electrode (CPE) and a CPE modified with different preparations of AgNO_3_ and/or solid silver particles. The chloride ion sensing characteristics were investigated. The CPE modified with silver particles promoted the sensitivity for chloride ions. Patil et al. [19] integrated pH, turbidity and temperature-sensing devices, in addition to global system for mobile communications (GSM), to investigate sensing characteristics and applications at different temperature conditions.

Graphenes are 2-D structures, providing large surface area, zero band gap, extremely high intrinsic charge carrier mobility and high chemical stability [20,21,22,23,24,25]. Many researchers have studied the physical characteristics of graphene [26,27]. Graphene was used to modify the ion sensors, with significant improvement of the sensing characteristics. Recently, Ali et al. [28] used graphene oxide (GO) nanosheets and poly(3,4-ethylenedioxythiophene) nanofibers (PEDOT-NFs) as electrochemical sensing interfaces to prepare microfluidic impedimetric nitrate sensors. They used electrochemical impedance spectroscopy (EIS), scanning electron microscopy (SEM) and transmission electron microscopy (TEM) to investigate the sensing characteristics of sensing film.

Our research group [29] investigated the sensitivity variation at the different weight ratios of the GO solution that were used to modify the arrayed flexible RuO_2_ chloride sensor. By adding GO, the sensitivity was enhanced, and this is attributed to the increased area of the sensing windows. In this study, temperature affected chloride sensors, therefore we investigated the sensitivities, drift effects and electrochemical impedance analysis of the arrayed flexible RuO_2_/GO chloride sensor by varying temperatures of the NaCl solution from 10 °C to 50 °C.

## 2. Materials and Methods

### 2.1. Materials

The flexible and light polyethylene terephthalate (PET) substrate was purchased from Zencatec Corporation (New Taipei, Taiwan). The silver paste and epoxy thermosetting polymer (product no. JA643) were used to prepare the conducting wires and insulation layer by a screen printing system. The silver paste and epoxy thermosetting polymer were purchased from Advanced Electronic Material Inc. (Tainan, Taiwan) and Everwide Chemical Co., Ltd. (Yunlin, Taiwan), respectively. The ruthenium target (Ru, 99.95 wt %) was used to deposit the thin ruthenium dioxide (RuO_2_) film onto the silver paste layer using a radio frequency sputtering system. The ruthenium target was purchased from Ultimate Materials Technology Co., Ltd. (Hsinchu, Taiwan). The graphene oxide powder was purchased from Tokyo Chemical Industry Co., Ltd. (Chuo-ku, Tokyo, Japan). The ETH9033 and TDDMACl were used as chloride sensing film, and they were purchased from Sigma-Alorich Co. Ltd. (St. Louis, MO, USA). Sodium chloride (NaCl) powder was purchased from Avantor Performance Materials, Inc. (Center Valley, PA, USA), and was then used to prepare the aqueous solutions.

### 2.2. Fabrication of the Arrayed Flexible RuO_2_/GO Chloride Ion Sensor

**Figure 2:**
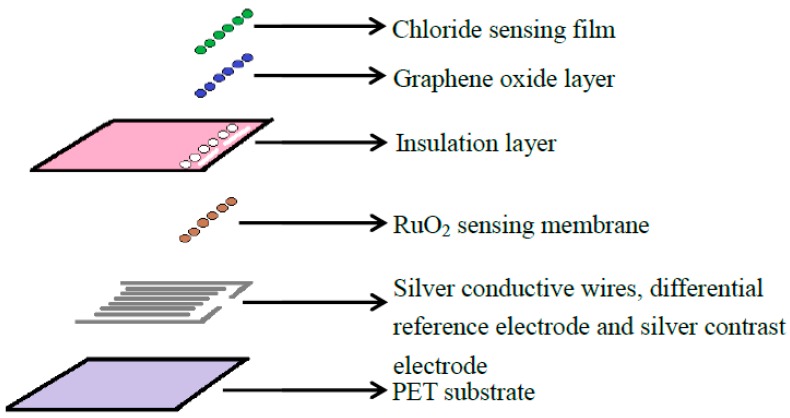
The fabrication process of the arrayed flexible RuO_2_/GO chloride ion sensor [14,29,30].

The fabrication process for the arrayed flexible RuO_2_/GO chloride ion sensor was shown in Figure 2. We used radio frequency sputtering and screen printing technology to fabricate the arrayed flexible RuO_2_ pH sensor [14,29,30]. The sensing area of RuO_2_ electrode is 1 mm × 1 mm. The 0.01 wt % GO solution was prepared with 10 mL deionized water and 1 mg graphene oxide powder, and the GO solution was uniformly mixed by ultrasonic vibration. Then we pipetted 2 μL of the 0.01 wt % GO solution onto each of the six sensing windows of the arrayed flexible RuO_2_ sensor. We then put the sensors on a table at room temperature (25 °C) for 12 h.

The weight ratios of the poly (vinyl chloride) (PVC), bis (2-ethylhexyl) sebacate (DOS), chloride ionophore III (ETH9033) and TDDMACl were 33:66:2:10 (wt %). The tetrahydrofuran (THF) solution was a solvent. The THF solution was used to prepare the chloride sensing mixture.

The 0.165 g of poly (vinyl chloride) (PVC) powder, 0.33 g of bis (2-ethylhexyl) sebacate (DOS) powder and 2.5 mL of tetrahydrofuran (THF) solution were uniformly mixed by the micromixer (Finemixer SH2000, Finepcr Corporation, Korea). The PVC, DOS and THF compounds comprised solution A.

The 5 mg of the ETH9033 powder and 0.5 mL of THF solution were uniformly mixed by the micromixer. The ETH9033 and THF compounds comprised solution B.

The 0.25 g of the TDDMACl and 1.25 mL of THF solution were uniformly mixed by the micromixer. The TDDMACl and THF compounds were solution C.

The chloride sensing mixture was composition of 20 μL of the solution A, 8 μL of the solution B and 2 μL of solution C. The chloride sensing mixture was uniformly mixed by the micromixer. Finally, we pipetted 2 μL of the chloride sensing mixture onto each of the six sensing windows of the arrayed flexible RuO_2_ sensor. Finally, the arrayed flexible RuO_2_/GO chloride sensors were dried at room temperature (25 °C) for 4 days.

Adjustable volume micropipettes (SIS-825.0020-1PAK, Socorex Isba S.A., Switzerland) were used to pipette 2 μL of 0.01 wt % GO solution and 2 μL of the chloride sensing mixture onto each of the six sensing windows of the sensors. We used the adjustable volume micropipettes to control the reproducibility of the mixture pipetting and thickness of these layers.

### 2.3. Sensing Mechanism of the Chloride Sensor

**Figure 3:**
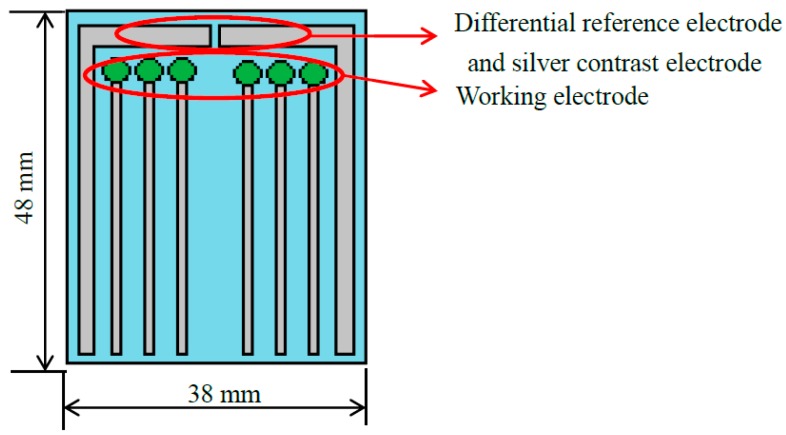
The schematic diagram of the arrayed flexible RuO_2_/GO chloride sensor [14].

**Figure 4:**
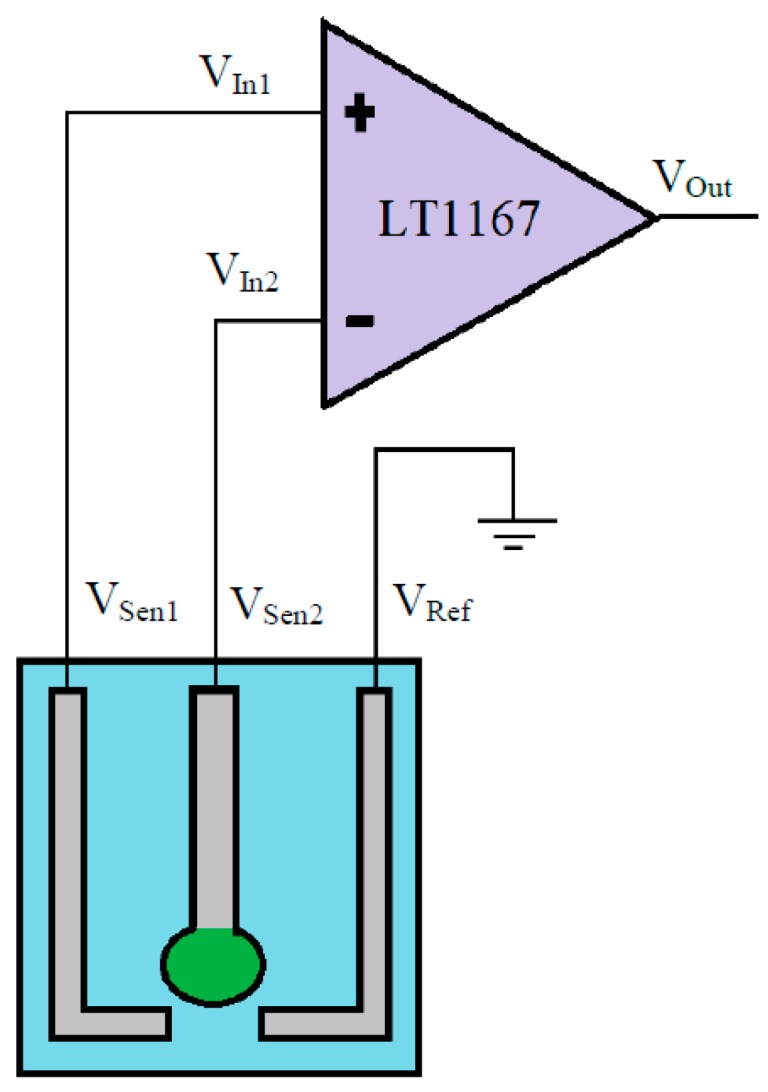
The schematic diagram of V-T measurement system with LT1167 instrumentation amplifier for the arrayed flexible RuO_2_/GO chloride sensor [30].

We used the screen printing system and silver paste to fabricate the difference reference electrodes and silver contrast electrodes, as shown in Figure 3. The voltage-time measurement system for the arrayed flexible RuO_2_/GO chloride sensor was shown in Figure 4. From Equation (2), the sensing mechanism of the single working electrode, the difference reference electrodes and silver contrast electrodes [30]. *V*_Out_ is the output potential of an LT 1167 amplifier, *V*_Ref_ is the potential of the silver reference electrode, *V*_Sen1_ is the potential of the silver contrast electrode, *V*_Sen2_ is the potential of the working electrode (sensing membrane), *V*_In1_ is the potential difference between the working electrode and the reference electrode and *V*_In2_ is the potential difference between the silver contrast electrode and the reference electrode. The Nersntian equation of the chloride sensing membrane was as shown in Equation (3). *E* is the electromotive force (EMF), *E*_0_ is the initial voltage, α is the activity of the ion, R is the gas constant 8.316 mol·e^−1^·°C^−1^, F is Faraday coefficient 96.487 °C. The response potentials were decreased when chloride concentration increased.

(2) $$V_{\mathrm{Out}}=V_{\mathrm{In}1}-V_{\mathrm{In}2}=\left(V_{\mathrm{Sen}1}-V_{\mathrm{Ref}}\right)-\left(V_{\mathrm{Sen}2}-V_{\mathrm{Ref}}\right)=V_{\mathrm{Sen}1}-V_{\mathrm{Sen}2}$$

(3) $$E=E_{0}-2.303\frac{RT}{F}\;\log\alpha =E_{0}-0.05916\;\mathrm{pCl}$$

### 2.4. Voltage-Time and Eelectrochemical Impedance Spectroscopy Measurement Systems

The power supply, National Instruments Data Acquisition (DAQ) card, readout circuit, arrayed flexible RuO_2_/GO chloride sensor and computer were integrated to compose a voltage-time (V-T) measurement system. We used the eight amplifiers (LT1167), core wires and a circuit board for the readout circuit. The voltage-time curves reflect the response potentials of different chloride concentrations from 1 ×10^−5^ M to 1 M NaCl solutions.

Electrochemical impedance spectroscopy (EIS; BioLogic SP 150, Aurora Biotech Inc., Seyssinet-Pariset, France) was used to get the solution resistance (R_s_), electron transfer resistances (R_et_) and double layer capacitor (C_dl_) between the sensing membrane and NaCl solution. The working electrode was an RuO_2_/GO/chloride ion sensing film, the reference electrode was an Ag/AgCl electrode and the counter electrode was a platinum (Pt) electrode. The amplitude of the voltage of the EIS measuring system was 0.7 mV, and the frequency range of the sinusoidal excitation signal was set from 100 MHz to 10 kHz in the EIS measuring system. The cooling circulating water bath and thermometer were used to control the solution temperatures from 10 °C to 50 °C, with concentrations from 1 × 10^−5^ M to 1 M NaCl.

The experiments of sensitivity, EIS and drift effect of the flexible arrayed RuO_2_/GO chloride sensor were described as follows:

1. The sensitivities were investigated from 1 × 10^−5^ M to 1 M NaCl solutions at room temperature (25 °C) with the V-T measuring system.
2. The sensitivities were investigated from 1 × 10^−5^ M to 1 M NaCl solutions at different temperatures from 10 °C to 50 °C with the V-T measuring system.
3. The electrochemical impedance analysis was used to measure and fit the values of R_et_, R_s_ and C_dl_ from 1 × 10^−5^ M to 1 M NaCl solutions at room temperature (25 °C) with the EIS measuring system.
4. The electrochemical impedance analysis was used to measure and fit the values of R_et_, R_s_ and C_dl_ in the 1 M NaCl solution at different temperatures from 10 °C to 50 °C with the EIS measuring system.
5. The response potential variations of 1 M NaCl solution were investigated over a longer period for different solution temperatures from 10 °C to 50 °C by the V-T measurement system.

Each experiment was tested five times and the average sensitivities, results of EIS analysis and drift rates were obtained.

## 3. Results and Discussion

### 3.1. Investigation of the Sensitivities for Different Solution Temperatures

**Figure 5:**
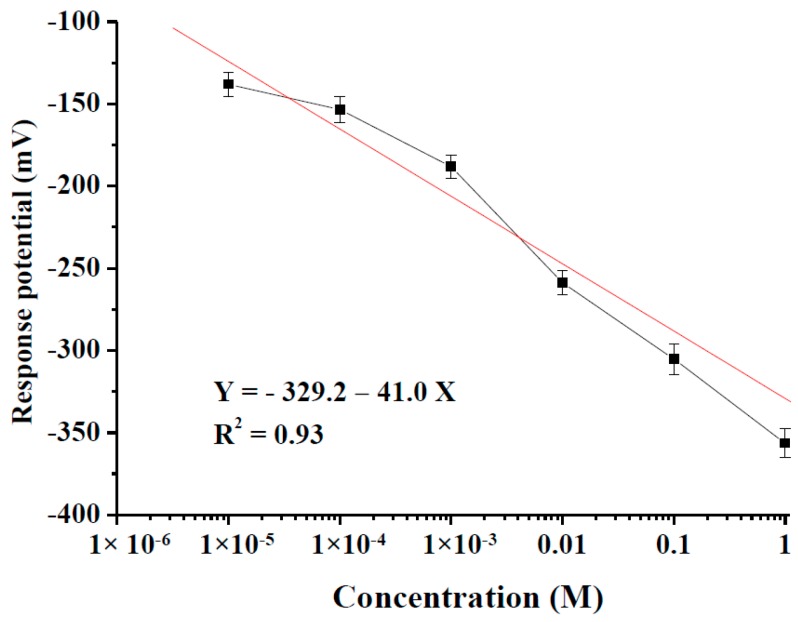
The curve of the response potential versus different chloride concentrations for the arrayed flexible RuO_2_/GO chloride sensor from 10^−5^ M to 1 M NaCl solutions at room temperature 25 °C.

In Figure 5, we can see the curves with the fitted parameters of Equations (4) and (5) as follows:

*Y* = −329.20 − 41.0*X* (4)

where *Y* is response potential and *X* is the log of chloride concentration.

*R*^2^ = 0.93 (5)

where *R*^2^ is the linearity of the curve.

The response potentials of the 1 × 10^−5^ M to 1 M NaCl solutions were −138.1 ± 7.5 mV (1 × 10^−5^ M), −153.4 ± 8.0 mV (1 × 10^−4^ M), −188.2 ± 7.1 mV (1 × 10^−3^ M), −258.8 ± 7.4 mV (0.01 M), −305.4 ± 9.3 mV (0.1 M) and −356.4 ± 8.8 mV (1 M). The average response potentials of the RuO_2_/GO arrayed flexible chloride ion sensors rose with chloride ion concentration. The GO contains the hydroxyl (–OH) and carboxyl (–COOH) groups. Protonation and de-protonation of –OH and –COOH groups accompany the pH variations [17,18,19,20,21,22]. Melai et al. [31] and Kim et al. [32] found the oxygen-containing functional groups base on the basal plane and edges of the GO structure. The oxygen-containing functional groups have negative ions. GO has large specific surface area and GO electrochemistry characteristics [29,31,32,33,34] improve the chloride sensitivity of RuO_2_/GO arrayed flexible chloride ion sensors. From Table 1, the average sensitivity of RuO_2_ arrayed flexible chloride ion sensors was 25.1 ± 11.3 mV/pCl at room temperature [14]. Dam et al. [35] used the screen printing system and Dupont 5876 AgCl conducting paste to prepare an AgCl layer on a PET substrate, which is a potentiometric sensing device. The sensitivity of the flexible chloride sensor was 57.0 mV/decade from 1 × 10^−3^ M to 3 M KCl solutions. Harris et al. [36] used the screen printing system and silver paste to prepare a silver layer on an alumina substrate, which is a potentiometric sensing device. The chloride sensors and distributed wireless network were used to detect chloride range. The sensitivity of the wireless chloride sensor was 59.2 mV/pCl from 62.5 × 10^−3^ M to 1 M NaCl solutions. Trnkova et al. [18] used the 70% graphite powder, 30% mineral oil, to fabricate the carbon paste electrode, which is an amperometric sensing device. The sensitivity of the carbon paste electrode was 1.1 nA/µM form 1 × 10^−4^ M to 1 × 10^−3^ M NaCl solutions. The sensitivities of their sensors were higher than the arrayed flexible RuO_2_/GO chloride sensor, but they used Ag/AgCl reference electrodes. We used the screen printing system and silver paste to fabricate the differential reference electrode and silver contrast electrode. The advantages of the arrayed flexible RuO_2_/GO chloride sensor are light weight, flexibility and low cost [14].

**Figure 6:**
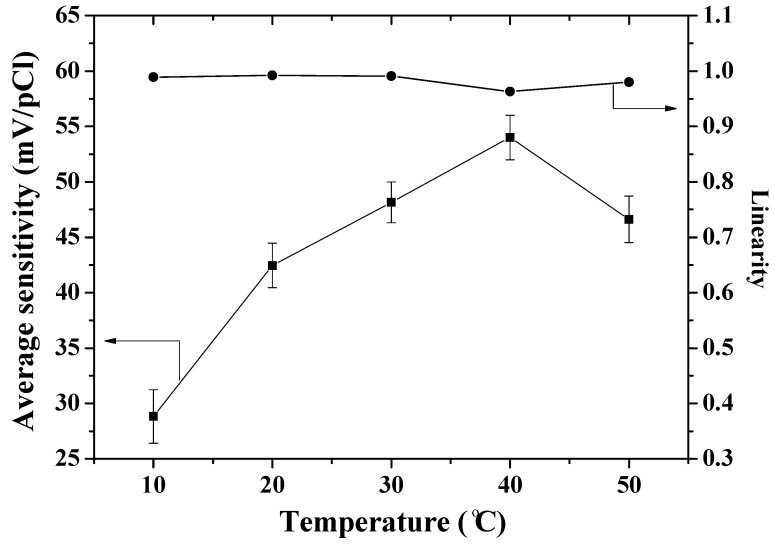
The average sensitivities and linearities of the different temperatures for the arrayed flexible RuO_2_/GO chloride sensor.

The sensing devices were used to take five measurements in NaCl solutions from 1 × 10^−5^ M to 1 M. The measured results are shown in Figure 6 and Table 2, where we see that the average sensitivities (absolute value) of the arrayed flexible RuO_2_/GO chloride sensors at different solution temperatures were 28.2 ± 2.4 mV/pCl (10 °C), 42.5 ± 2.0 mV/pCl (20 °C), 47.1 ± 1.8 mV/pCl (30 °C), 54.1 ± 2.0 mV/pCl (40 °C) and 46.6 ± 2.10mV/pCl (50 °C). According to the experimental results and our previous research [37], the average sensitivities of arrayed flexible RuO_2_/GO chloride sensors were higher than flexible RuO_2_ chloride sensors at different solution temperatures. GO has large specific surface area, which supported the chloride ion sensing film to obtain more chloride ions and produce the bigger response potentials than if not GO-modified.

**Figure 7:**
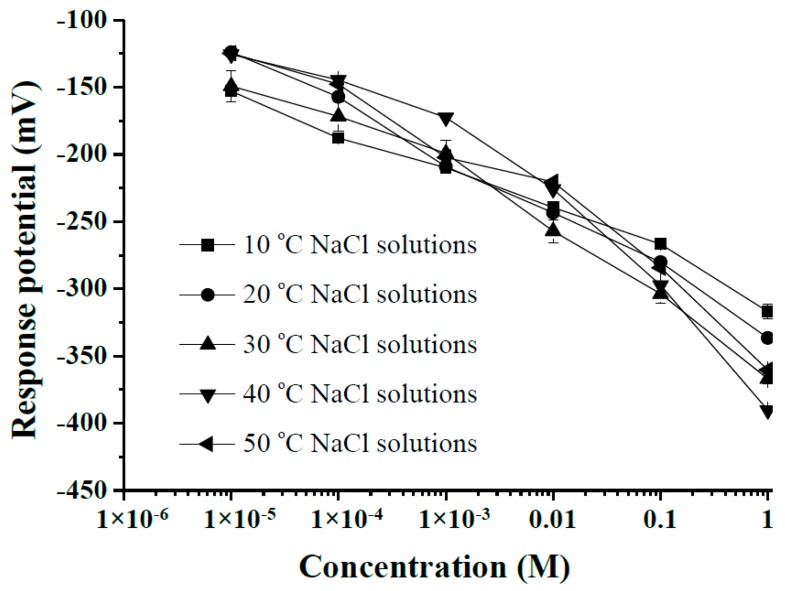
The response potentials of the arrayed flexible RuO_2_/GO chloride sensor at different NaCl solution temperatures from 10 °C to 50 °C.

From Figure 7, the response potentials for the 1 × 10^−5^ M solution from 10 °C to 50 °C were −151.1 ± 3.4 mV (10 °C), −124.1 ± 2.1 mV (20 °C), −156.0 ± 1.6 mV (30 °C), −125.7 ± 2.1 mV (40 °C) and −125.0 ± 2.1 mV (50 °C) mV. On the other hand, the response potentials for the 1 M solution from 10 °C to 50 °C were −319.7 ± 2.7 mV (10 °C), −336.3 ± 1.6 mV (20 °C), −369.1 ± 2.6 mV (30 °C), −390.3 ± 1.1 mV (40 °C) and −360.3 ± 2.1 mV (50 °C). The response potentials for the 1 M declined with NaCl temperature over 10–40 °C. The response potential differences of the 1 × 10^−5^ M and 1 M NaCl solutions rose with solution temperature over 10–40 °C.

### 3.2. Investigation of the Electrochemical Impedance Analysis for Different Solution Temperatures

**Figure 8:**
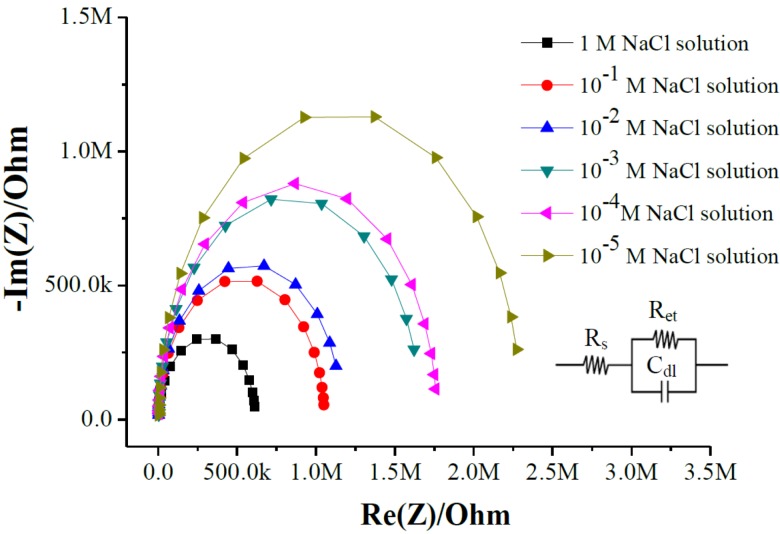
The electrochemical impedances of fitted curves at the different chloride ion concentrations.

From Figure 8 and Table 3, we see that the electron transfer resistances (R_et_) of the RuO_2_/GO arrayed flexible chloride ion sensors were decreased in NaCl solutions from 1 × 10^−5^ M to 1 M. The chloride ion sensing film caught the chloride ions at the different chloride ion concentrations from 1 × 10^−5^ M to 1 M NaCl solutions, which could transform to the response potentials at different chloride ion concentrations [10,13,14].

**Figure 9:**
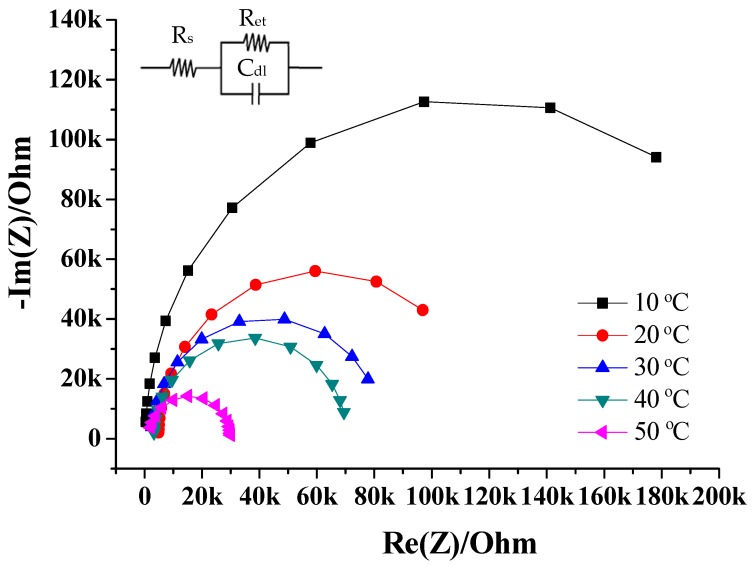
The electrochemical impedances of fitted curves in the different solution temperatures from 10 °C to 50 °C.

We used 1 M NaCl solution to investigate the R_et_ for different solution temperatures from 10 °C to 50 °C. From Figure 9 and Table 4, the R_et_ were 274.7 ± 52.7 kΩ (10 °C), 129.9 ± 25.1 kΩ (20 °C), 83.8 ± 4.3 kΩ (30 °C), 41.5 ± 13.0 kΩ (40 °C) and 34.9 ± 11.8 kΩ (50 °C).

At higher solution temperatures, the solution viscosity is lower and the mobility of the ions in solution is higher. The dissociation of molecules increases with solution temperature, which induced the number of ions in solution to increase with the conductivity of a solution [38,39], which helped the chloride film to catch an increasing amount of chlorides as the temperature of the NaCl solution was increased from 10 °C to 40 °C. However, the adhesion between the chloride ion sensing film and RuO_2_/GO sensing window was lower at 50 °C than at 40 °C, and the response potentials were also lower across the chloride concentrations at 50 °C. The average sensitivity rose with solution temperature over 10–40 °C, but was lower at 50 °C. The operating temperatures of the arrayed flexible RuO_2_/GO chloride sensor were from 10 °C to 40 °C. The temperature coefficient of sensitivity (TCS) of the arrayed flexible RuO_2_/GO chloride sensor was found to be approximately 0.81 mV/(pCl·°C).

### 3.3. Investigation of the Drift Effect at Different Solution Temperatures

We investigated the response potentials over a longer period in NaCl solution with different solution temperatures. The V-T measuring system was used to measure response potentials for the arrayed flexible RuO_2_/GO chloride sensor in the 1 M NaCl solution over 12 h across the 10 °C to 50 °C conditions. In Table 5 we see that the maximum and minimum drift rates were 8.2 mV/h and 2.5 mV/h at 10 °C and 50 °C, respectively. The RuO_2_/GO chloride ion sensing film produced a hydrated layer during measurement over a longer period [14,40] at room temperature, which caused the response potential to increase. Some researchers [7,41] used an RF sputtering system to prepare different metal oxides for a sensing membrane on the different substrates. They investigated the drift effects of their pH sensor at different solution temperatures. The drift variations were higher when the pH solution temperatures were higher. As per to Section 2.2, we pipetted the 2 μL of the chloride sensing mixture onto the six sensing windows of each sensor. The chloride sensing mixture was similar to the colloid. The chloride sensing films and sensing windows of the arrayed flexible RuO_2_/GO chloride sensors adhered to each other. The adhesion between the chloride ion sensing film and RuO_2_/GO sensing window was lower at higher temperatures. The lower adhesion caused the drift rate to decrease at higher temperatures (from 40 °C to 50 °C). The drift variations were declined with temperature of the 1 M NaCl for the 12 h treatment.

## 4. Conclusions

The average sensitivities of the arrayed flexible RuO_2_/GO chloride sensor were 28.2 ± 1.4, 42.5± 2.0, 47.1 ± 1.8, 54.1 ± 2.0 and 46.6 ± 2.1 mV/pCl with different concentrations of chloride solution at 10, 20, 30, 40 and 50 °C. The average sensitivities rose with solution temperature from 10 °C to 40 °C. The operating temperatures of the arrayed flexible RuO_2_/GO chloride sensor were from 10 °C to 40 °C. We found the drift effects of the arrayed flexible RuO_2_/GO chloride sensor in the 1 M NaCl solution to be between 8.2 mV/h and 2.5 mV/h with solution temperatures from 10 °C to 50 °C. The temperature coefficient of sensitivity (TCS) of the arrayed flexible RuO_2_/GO chloride sensor was approximately 0.81 mV/(pCl·°C).

## Figures and Tables

**Table 1:** The comparisons of the average sensitivity of the arrayed flexible RuO_2_/GO chloride sensor and other research on different chloride concentrations.

Sensing Film	Sensing Mechanism	Sensitivity (pCl)	Detection Chloride Range (M)	Reference
PET/RuO_2_/GO/chloride film	Potentiometric	41.0 mV/pCl	1 × 10^−5^ M to 1 M	In this study
PET/RuO_2_ chloride film	Potentiometric	25.1 mV/pCl	1 × 10^−5^ M to 1 M	[14] 2106
PET/AgCl paste	Potentiometric	57.0 mV/decade	1 × 10^−3^ M to 3 M	[35] 2015
Alumina/silver	Potentiometric	59.2 mV/pCl	6.25 × 10^−4^ M to 1 M	[36] 2016
Carbon paste electrode	Amperometric	1.1 nA/µM	1 × 10^−4^ M to 1 × 10^−3^ M	[18] 2008

**Table 2:** Comparison of the sensitivities of PET/RuO_2_/GO and PET/RuO_2_ [37] chloride sensor at different solution temperatures.

Solution Temperature (°C)	Sensitivity (mV/pCl)	
	PET/RuO_2_/GO (In This Study)	PET/RuO_2_ [37] 2017
10	28.2 ± 1.4	27.7 ± 0.0
20	42.5 ± 2.0	36.8 ± 0.0
30	47.1 ± 1.8	39.8 ± 1.3
40	54.1 ± 2.0	41.5 ± 1.6
50	46.6 ± 2.1	22.6 ± 0.0

**Table 3:** The fitted results of R_et_, R_s_ and C_dl_ of the arrayed flexible RuO_2_/GO chloride sensor at different NaCl concentrations solution from 1 × 10^−5^ M to 1 M.

NaCl Concentration (M)	R_et_ (kΩ)	R_s_ (kΩ)	C_dl_ (pF)
1	584.3 ± 30.7	3.5 ± 0.1	73.4 ± 0.8
0.1	1047.3 ± 6.4	2.7 ± 0.2	93.8 ± 0.8
1 × 10^−2^	1131.7 ± 24.8	2.6 ± 0.3	88.8 ± 0.2
1 × 10^−3^	1681.0 ± 32.9	3.4 ± 0.3	85.8 ± 2.7
1 × 10^−4^	1728.3 ± 44.2	4.6 ± 0.3	70.4 ± 1.0
1 × 10^−5^	2350.5 ± 71.4	3.3 ± 0.3	94.0 ± 0.8

**Table 4:** The fitted results of R_et_, R_s_ and C_dl_ in 1 M NaCl solution at the different solution temperatures.

Solution Temperature (°C)	Electron Transfer Resistance R_et_ (kΩ)	Solution Resistance R_s_ (kΩ)	Double Layer Capacitor C_dl_ (pF)
10	274.7 ± 52.7	0.4 ± 0.2	238 ± 37.6
20	129.9 ± 25.1	3.6 ± 1.8	535 ± 29.4
30	83.8 ± 4.3	2.5 ± 0.2	543 ± 21.0
40	41.5 ± 13.0	1.2 ± 1.3	339 ± 37.5
50	34.9 ± 11.8	2.1 ± 0.9	416 ± 10.2

**Table 5:** The drift rates of the arrayed flexible RuO_2_/GO chloride sensor in the 1 M NaCl solution from 10 to 50 °C.

Solution Temperature (°C)	Drift Rate (mV/h)
10	8.2
20	4.7
30	4.2
40	3.6
50	2.5
